# Impact of changed management policies on operating room efficiency

**DOI:** 10.1186/1472-6963-14-224

**Published:** 2014-05-20

**Authors:** Birgithe E Sandbaek, Berit I Helgheim, Odd I Larsen, Sigurd Fasting

**Affiliations:** 1 Faculty of Economics, Informatics and Social Sciences, Molde University College, Specialized University in Logistics, PB 2110, 6402 Molde, Norway; 2 Department of Anaesthesia & Intensive Care Medicine, St Olav University Hospital, Trondheim N-7006, Norway

**Keywords:** Dedicated operating room, Emergency surgery, Operating room efficiency, OR booking, Patient classification, Priority setting, Resource allocation

## Abstract

**Background:**

To increase operating room (OR) efficiency, a new resource allocation strategy, a new policy for patient urgency classification, and a new system for OR booking was implemented at a tertiary referral hospital. We investigated the impact of these interventions.

**Methods:**

We carried out a before-and-after study using OR data. A total of 23 515 elective (planned) and non-elective (unplanned) orthopaedic and general surgeries were conducted during calendar year 2007 (period 1) and July 2008 to July 2009 (period 2). The Wilcoxon–Mann–Whitney test was used to calculate statistical significance.

**Results:**

An increased amount of case time (7.1%, p < 0.05) was conducted without any increase in out-of-hours case time. Despite having three fewer ORs for electives, slightly more elective case time was handled with 26% less use of overtime (p < 0.05). Mean OR utilization was 56% for the 17 mixed ORs, 60% for the 14 elective ORs, and 62% for the 3 dedicated ORs. A 20% growth (p < 0.05) of non-elective case time was primarily absorbed through enhanced daytime surgery, which increased over 48% (p < 0.05). As a result, the proportions of case time on evenings and nights decreased. Specifically, case time at night decreased by 26% (p < 0.05), and the number of nights without surgery increased from 55 to 112 (out of 315 and 316, respectively). Median waiting time for the middle urgencies increased with 1.2 hours, but over 90% received treatment within maximum acceptable waiting time (MAWT) in both periods. Median waiting time for the lowest urgencies was reduced with 12 hours, and the proportion of cases treated within MAWT increased from 70% to 89%. The proportion of high urgency patients (as a proportion of the total) was reduced from 20% to 12%. Consequently, almost 90% of the operations could be planned at least 24 hours in advance.

**Conclusions:**

The redesign facilitated effective daytime surgery and a more selective use of the ORs for high urgency patients out of hours. The synergistic effect probably exceeded the sum of the individual effects of the changes, because the effects of each intervention facilitated the successful implementation of others.

## Background

Currently, limited health care resources face almost limitless demands. The application of advanced planning and scheduling tools is therefore becoming increasingly important in hospitals. As well as choosing an appropriate way to allocate resources to meet different medical priorities, it is also necessary to increase operating room (OR) efficiency. In this paper, the term “OR efficiency” refers to maximizing throughput and OR utilization while minimizing overtime and waiting time, without additional resources.

To increase OR efficiency and facilitate effective daytime surgery and a more selective use of the ORs for high urgency patients out of hours, a series of interventions were implemented at a major tertiary referral hospital. The redesign involved a change in allocation strategy from mixed to dedicated resources, and new policies for patient urgency classification and booking of ORs for elective (planned) and non-elective (emergency/unplanned) orthopaedic and general surgery patients.

### To dedicate or not to dedicate

OR efficiency in hospitals with a mixed caseload of elective and non-elective patients is strongly influenced by the stochastic arrival of non-elective patients. To deal with the underlying uncertainty, there are two common ways of allocating OR capacity for non-elective patients. The first alternative is the “mixed” policy, which reserves some capacity for non-electives in all ORs. The other is to isolate the variability caused by non-elective work, and “dedicate” particular ORs to that work, leaving others exclusively for elective work. These allocation problems are not new, and hospitals vary in their approach. Recently published guidelines recommend a separation of elective and non-elective work [1,2]. In the literature, however, there is no consensus on the issue. It can be argued that separating planned and unplanned surgery can reduce total variability, increase predictability, and improve responsiveness and overall productivity [3,4]. This is supported by empirical studies comparing efficiency after a change in allocation strategy [5-7], and additional changes in management policies such as patient classification systems [8,9]. Simulation studies, however, indicate that such a separation is less cost-effective and increases non-elective response time [10-12]. These studies conclude that insertion of “slack” in all ORs provides more flexibility, allowing them to manage variation in non-elective volumes and case duration, whilst a dedicated policy ties up capacity whether there are emergency patients or not. While some research has addressed the impact of these changes on OR efficiency, most has focused on the isolated effects of a single strategy change, such as one dedicated OR for a single department, or has used a simulation model. Although one recent study focused on both elective and non-elective patients [6], most studies have covered only one or two patient groups, usually either elective or non-elective. None of them, however, investigated the efficiency impact of several interventions including the introduction of three dedicated ORs. By distributing non-elective variability over three ORs, higher flexibility is offered than having just one dedicated OR.

### Factors influencing the distribution of patient categories

Pre-operative categorization (triaging) of surgical patients is the process of defining their level of urgency and scheduling their surgery accordingly. The categories define the urgency, for example (i) elective patients (within weeks or months) and (ii) non-elective patients. The latter group is divided into three categories in our hospital: within 6 hours (U1), within 24 hours (U2), and within 72 hours (U3). Interventions affecting patient classification and OR booking may influence the prioritization process and can therefore alter the distribution of patient categories. Currently, there is no agreement on any level (strategic, tactical and operational) of health systems about which values should guide the decisions for priority-setting [13]. Existing definitions of emergency surgery are not standardized across different jurisdictions or countries, or even across hospitals and departments [14]. Fitzgerald et al. [15] conducted a survey asking decision-makers about the urgency of a set of clinical conditions and appropriate waiting times for the patients. Their findings indicated that discrepancies around urgency classifications and acceptable timeframes for treatment can cause variability in the assignment of urgency for patients with similar conditions. The urgency of an intervention is not a constant factor. With the exception of time-critical emergencies, where delays may put life, organs, or limbs at risk, the decision-making processes around the prioritization and scheduling of surgery are not based only on clinical evaluation. They are also influenced by non-clinical differentiators, such as logistical constraints (e.g. availability of resources and time), conflicting objectives, disparities in the perception of urgency, and competing priorities [15]. Cases can be given a higher priority to secure an earlier slot (“gaming”) [16], or requests for surgery can be postponed to avoid cancellation of an elective case (“delayed booking”). The clinical urgency is therefore not necessarily identical to the level of priority given. Prioritization dynamics are affected by several mechanisms, for example by strategic decisions such as the current reimbursement system or guaranteed maximum waiting times for elective cases*.* By rewarding high volumes of surgeries, the system provides financial incentives for competition between departments to ensure their own profitability, providing additional incentives for non-clinical prioritization. Although the concept of consistent prioritization and the development of standardized categories of clinical urgency have been discussed in the literature, there is a lack of research on this topic [14]. As far as we know, there have been no empirical investigations into the quantitative impact on the distribution of patient categories of dedicated ORs and new policies on pre-operative categorization (triaging) and OR booking.

To investigate the impact of the interventions, we carried out a before-and-after study using OR data to examine the volumes and case times by population and work-shift, as well as elective overtime, mean OR utilization, and non-elective waiting time. We then assessed the quantitative impact on the distribution of patient categories, and the distribution of case time by shift for the three non-elective categories.

## Methods

### The setting

St Olav’s Hospital is a publicly-financed tertiary referral hospital that provides both elective and non-elective surgery. Its activities also include education and research. It is a local hospital for a population of 275 000 and has several regional and national roles for the population in three nearby counties, with a total of 630 000 inhabitants. Emergencies cannot be diverted and treated elsewhere because there are no alternative hospitals nearby. Until 2008, the hospital employed a mixed policy, but a dedicated policy was then adopted. The catchment population did not change.

### The problem

Common problems in the ORs included delays and disruptions in the planned schedule of surgery. Non-elective cases were inserted into slots between planned surgery resulting in postponements or cancellation of electives and staff working overtime. Alternatively, to avoid disrupting the planned schedule, lower urgencies were postponed until the evening or night when resources were limited. However, these “overflow” cases could lead to cancellations the next day, because of a shortage of available beds in the post anaesthesia care unit, or because the postponed case could bump another patient on the next day’s schedule. To avoid this, the deferred patients frequently suffered on-going delays following prioritisation of the next day’s non-elective list.

### The redesign

A series of interventions was implemented during the first half of 2008 to change the way that orthopaedic and general surgery was managed. The redesign was initiated and implemented by the process owners (physician and nurse team leaders). First, a dedicated policy was adopted. During the dayshifts, 3 staffed ORs were dedicated to non-elective surgery, and the 14 remaining ORs were used for elective surgery. The total number of available ORs remained unchanged. Determination of the required number of dedicated ORs was based on historical demand (i.e. mean non-elective case time). The standby resources for emergencies during evening and night were the same in both periods. Second, to ensure consistent patient classification and OR booking, new policies were implemented. Before the reforms, non-elective cases could be booked as soon as the need for surgery had been identified. Afterwards, requests could not be sent until the patient was ready for surgery. The patient classification system, originally based on the individual doctor’s evaluation, was replaced by a consensus-based standardized system developed by the surgeons. Criteria for the different urgencies were provided as diagnoses pre-assigned with urgency levels/colour codes: U1 (red), U2 (yellow), and U3 (green). The time frame for each category remained the same. U1 cases were further sub-divided by the surgeon who posted the case, into immediately, within minutes, or within hours. Clinical evaluation would, if necessary, overrule the predetermined levels, thereby ensuring flexibility to recognize the dynamics of an individual patient’s clinical condition. To facilitate implementation, the system was made transparent to enable collegial assessment of compliance.

### The data

The OR data included all elective and non-elective orthopaedic and general surgery cases and were collected by OR nurses at the time of surgery. They included the urgency levels, the dates and times for OR booking for non-electives, and OR entry and exit times. Case time was defined as the time between OR entry and exit. Waiting time was the time between OR booking and OR entry. We defined the shifts as follows: the day shift ran from 7 am to 4 pm, the evening shift from 4 pm to midnight, and the night shift from midnight to 7 am. Out-of-hours or overtime was 4 pm to 7 am during weekdays, and anytime at weekends.

Mean OR utilization was defined as the ratio between total case time and total available time. The data did not contain sufficient information to distinguish between the different ORs. Period 1 was calendar year 2007, and period 2 was the two last quarters of 2008 and the two first quarters of 2009. The first two quarters of 2008 were considered to be a transitional period that was therefore unsuitable for analysis. The data included 25 473 surgical cases performed in the two periods. After the exclusion of seven low-activity weeks in each period (Christmas, Easter, and summer vacation) and removal of cases with coding errors such as OR exit before surgical end time, the final dataset consisted of 23 515 orthopaedic and general surgeries. The individual observations were aggregated by day. The total number of days included in the final dataset was 315 and 316 days in the two periods. The Wilcoxon-Mann-Whitney test was used to calculate statistical significance. However, as an excessive number of zeros compromises its power [17], it was unsuitable for testing the significance of U1, U2 and U3 case times on the different shifts. Additionally, when assessing the case times on each shift by urgency level, the distributions were severely skewed with different shapes for each period, and no simple analytic function (transformation) provided an adequate fit. We did not, therefore, apply additional methods such as statistical analysis of censored data.

This paper is part of a research project between Molde University College and St Olav’s University Hospital, Trondheim. Permission to use the data was granted by the hospital’s steering group for acute surgery. The data were extracted from routine data recordings from every surgical procedure in the time period. The project was reviewed by the regional committee for Medical and Health Research Ethics, REC Central, who waived the need for approval.

## Results

The descriptive statistics are displayed in Table 1. Total number of patients increased by 6.8%, p < 0.05 (from 11 372 to 12 143). Total case time increased by 7.1%, p < 0.05, but out-of-hours case time did not change significantly (3, 2%).

**Table 1:** Descriptive statistics, total population and the two main patient groups

**Population**	**Variable**	**Period 1**	**Period 2**	**Change, %**
**Total**	Number of days (n)	315	316	
	Number of patients (n)	11 372	12 143	6.8*
	Case time (hours)	23 532	25 202	7.1*
	Out-hours operating (hours)	5 236	5 400	3.2
**Elective**	Number of patients (n)	7 709	8 044	4.3*
	Case time (hours)	16 433	16 693	1.6
	Overtime (hours)	494	367	−26*
	SD case time per day	1 509	1 227	
	Number of operating rooms (n)	17	14	
**Non - elective**	Number of patients (n)	3 663	4 099	12*
	Case time (hours)	7 099	8 509	20*
	Case time day (hours)	2 534	3 763	48.5*
	Case time evening (hours)	2 156	2 337	8.4*
	Case time night (hours)	582	440	−24.4*
	Case time weekend (hours)	1 827	1 969	7.8
	Day shift, percentage of total case time (%)	36	44	
	Evening shift, percentage of total case time (%)	30	27	
	Night shift, percentage of total case time (%)	8	5	
	Weekend, percentage of total case time (%)	26	23	
	Number of nights without *s*urgery (n)	55	112	
	SD daily case time (dayshift)	352	281	
**Total**	Mean utilization 17 OR s (%)	56	-	
**Elective**	Mean utilization 14 OR s (%)	-	60	
**Non - elective**	Mean utilization 3 OR s (%)	-	62	
	Mean utilization evening (%)	48	52	
	Mean utilization night (%)	37	28	

Note. *p<0.05. SD = Standard Deviation. Calculations of mean OR utilization on dayshifts include weekdays with elective surgery (n = 221 and 220 in period one and two, respectively). Consequently, a total of 40 hours (period one) and 74 hours (period two) non-elective case time conducted on dayshifts without elective cases is excluded. Mean OR utilization on evenings and nights include all weekdays in the two periods; 225 days and 226 weekdays.

### Elective and non-elective patient groups

**Figure 1:**
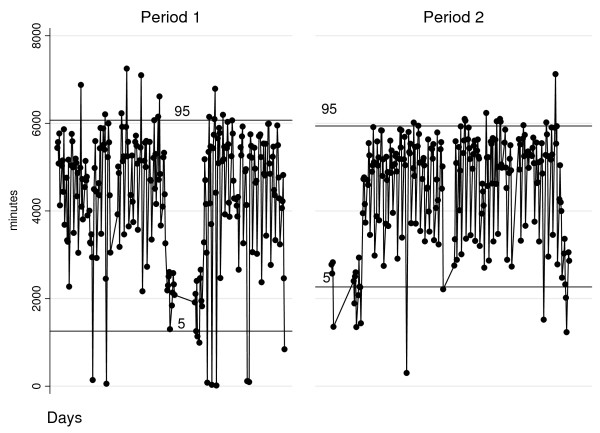
**Daily elective case time, two-way graph.** Each circle corresponds to one day; the lines indicate the 5th and 95th percentiles; the areas without observations represent low-activity periods, such as Public Holidays and vacations.

The number of ORs available for electives was reduced from 17 to 14 in the second period, because three ORs were dedicated to non-elective work. While there was a 4.3% increase in elective cases (335 patients, p < 0.05), there was no significant change in elective case time. Elective overtime was reduced by 26%, p < 0.05. Additionally, there was a reduction in daily elective case time variation; the standard deviation decreased from 1 509 to 1 227 (Figure 1).

Non-elective cases and case time increased by 12% and 20%, respectively (p < 0.05). Although, there was a 48% increase in case time on dayshifts and an 8.4% increase during the evenings, case time during nightshifts was reduced by over 24% (p < 0.05). At the same time, the number of nights without surgery increased from 55 to 112. As a result, non-elective work was largely carried out during dayshifts in the second period, this proportion of case time increased from 36% to 44%. The proportions of surgery taking place at evening and night decreased from 30% to 27% and from 8% to 5%, respectively. The proportion of surgery taking place at weekends did not change significantly.

Mean OR utilization was 56% for the 17 mixed ORs, 60% for the 14 elective ORs and 62% for the 3 dedicated ORs (Table 1). While mean OR utilization during evenings increased from 48% to 52%, there was a reduction from 37% to 28% during nights.

**Figure 2:**
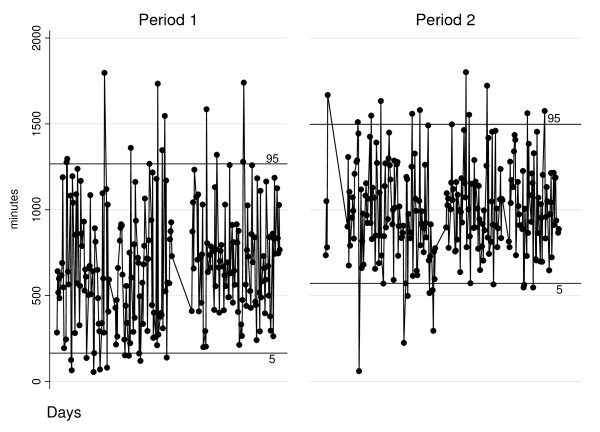
**Daily non-elective case time (day shifts, weekdays), two-way graph.** Each circle corresponds to one day shift; the lines indicate the 5th and 95th percentiles; the areas without observations represent low-activity periods, such as Public Holidays and vacations.

There was a reduction in non-elective daily case time variation on dayshifts, with the standard deviation reduced from 352 to 281 in the second period (Figure 2). The discrepancy between numbers of cases and case times for elective and non-elective work indicates a shift to surgeries with different durations.

### The non-elective categories

The changes in volumes and case times were differently distributed for the three non-elective categories (Table 2). There was a 36% decrease in both number of U1 cases and case time (p < 0.05). U2 and U3 cases increased 92% and 90% (p < 0.05). The corresponding case times increased by 96% and 105% (p < 0.05). For both groups, the increased case time was distributed over 47 additional days, because a larger number of the available days was used for these cases following the changes.

**Table 2:** Descriptive statistics, non-elective patient categories

**Population**	**Variable**	**Period 1**	**Period 2**	**Change, %**	**Diff. (P2-P1)**
**U1**	Number of patients (n)	2276	1448	−36*	
	Case time (hours)	4235	2726	−36*	
	Number of days with surgery	314	308		
	Percentage of patients treated within MAWT (%)	63	76		
	Median waiting time (hours)	3.2	1.7		−1.5*
**U2**	Number of patients (n)	609	1171	92*	
	Case time (hours)	1106	2171	96*	
	Number of days with surgery	249	296		
	Percentage of patients treated within MAWT (%)	97	93		
	Median waiting time (hours)	4	5		1*
**U3**	Number of patients (n)	778	1480	90*	
	Case time (hours)	1758	3612	105*	
	Number of days with surgery	261	308		
	Percentage of patients treated within MAWT (%)	70	89		
	Median waiting time (hours)	35	23		−12*

Note. *p<0.05. Diff. = Difference (period 2 - period 1); MAWT = maximum acceptable waiting time; Changes in waiting time between the two periods are compared using the Mann–Whitney U-test. It should be noted that waiting times analysis use disaggregate (patient level) data, while case times analysis use data aggregate per day (i.e.one observation corresponds to one day).Medians are reported because the distributions are skewed by cases with excessive waiting, resulting in mean waiting time being profoundly affected.

There was a discrepancy between the growth in number of U3 cases and case time. Despite the large reduction in U1 cases, there was a 20% growth of non-elective cases, solely from cases with lower urgency.

### The distribution of patient categories

**Figure 3:**
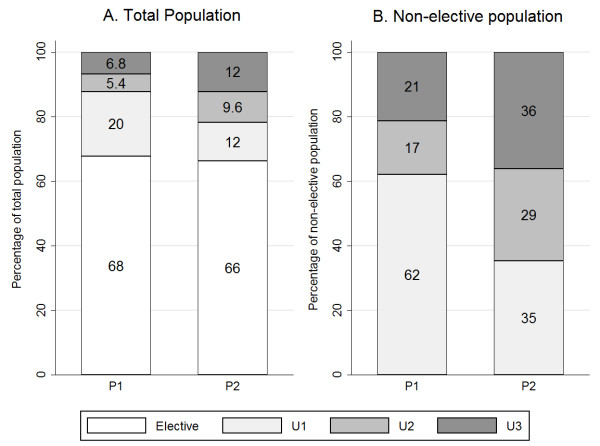
**Percentage distribution of patient categories by population. A**: Elective, U1, U2 and U3 percentages of total population. **B**: U1, U2 and U3 percentages of the non-elective population.

Figure 3 illustrates the percentage distributions of the patient categories in the total-, and non-elective populations. The elective group was by far the largest, 68% and 66% in periods one and two, respectively. Although the percentage ratio between the elective and non-elective groups remained almost the same, the distribution of the non-elective categories changed substantially. With the large reduction in U1 cases and the growth of U2 and U3 cases, more non-elective patients (65%) were U2 and U3 cases in the second period. U1 cases accounted for 20% of the total population in the first period, but only 12% in the second, and so almost 90% of the patients could be planned at least 24 hours in advance. Almost 80% of the patients, that is, all elective (66%) and U3 (12%) patients, could be planned at least 72 hours in advance. These changes provided huge benefits for planning and scheduling.

### The distribution of U1, U2 and U3 case time by shift

**Figure 4:**
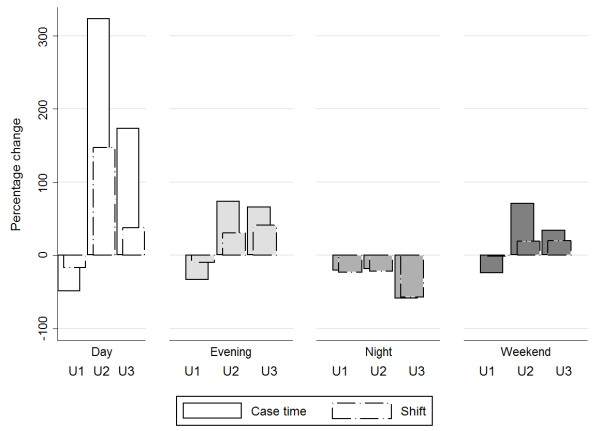
Percentage change of U1, U2 and U3 case time by shift, and number of day-, evening-, night-, and weekend shifts with surgery.

The examination of case time by shift for each non-elective category showed major changes (Figure 4). For example, U2 case time on dayshifts increased from 149 hours to 630 hours, a growth of more than 300%. This was possible because the number of dayshifts including U2 surgeries increased by 147% (from 66 to 163 shifts).

**Figure 5:**
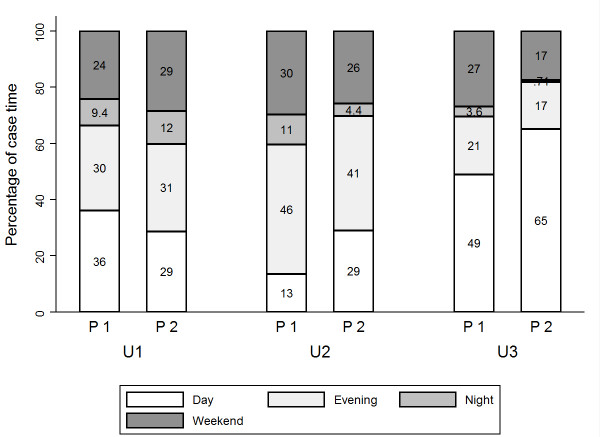
Percentage distribution of total U1, U2 and U3 case time by shift.

These changes were differently distributed for the three categories. We therefore examined the percentage distribution of case time by shift for each patient category in the two periods (Figure 5). In the first period, 36% of the total U1 case time was conducted during dayshifts. This was reduced to 29% in the second period, as a higher proportion of U1 work was completed during evenings, nights and weekends. U2 and U3 work, on the other hand, was largely carried out during dayshifts in the second period. In the first period, 13% of total U2 case time was handled during dayshifts. In the second period, this increased to 29%. The proportion of U3 case time on dayshifts increased from 49% to 65%. Unlike U1, the proportion of out-of-hours surgery was reduced for U2 and U3 cases. For U2 work, the proportion of case time during evening shifts decreased from 46% to 41%, and at night from 11% to 4.4%. For U3 work, these proportions decreased from 21% to 17%, and 3.6% to 0.7%. The proportion of surgery taking place at weekends was also reduced for both groups. These findings indicate that more effective daytime surgery absorbed this increase in U2 and U3 work.

### Waiting time

The proportion of U1 cases treated within maximum acceptable waiting time (MAWT) increased from 63% to 76%, and median waiting was reduced from 3.2 hours to 1.7 hours (Table 2). Median U2 waiting times increased from 3.8 hours to 5 hours, but over 90% of the patients received treatment in both periods. The proportion of U3 cases treated within MAWT increased from 70.4% to 89%, and median U3 waiting was reduced from 35 hours to 23 hours.

## Discussion

This study documents that the interventions improved OR efficiency in terms of throughput and OR utilization as well as overtime and waiting time, without additional resources. An increased amount of case time (7.1%, p < 0.05) was conducted without any increase in out-of-hours case time. As the catchment population did not change between the two periods, we can assume that there was a similar case-mix.

### Elective patients

Despite the reduction in the number of ORs available for elective work, slightly more elective case time was handled, with 26% (p < 0.05) less overtime. Mean OR utilization increased from 56% in period 1 to 60% in period two. These findings indicate that the 14 elective ORs were able to plan and operate more efficiently, avoiding “slack time” previously caused by insertion of non-elective cases in the mixed ORs. Daily case time variability for electives also decreased, indicating less disturbance from non-elective cases.

Our data contain no information on postponements of elective surgery or delays inflicted on elective patients in either period. This type of information might have indicated additional benefits. An advantage of separating elective and non-elective operations is that this provides the opportunity to establish “fast-track ORs”, which can further improve OR efficiency.

### Non-elective patients

Interpretation of the non-elective results is complicated by the number of changes. The effects could be from any one of the interventions. For example, the new patient classification system might have had an impact on efficiency even without the introduction of the dedicated ORs. The reclassification of cases that would previously have been U1 to lower levels of urgency caused a distributional shift, meaning that surgery for almost 90% of all patients could be planned at least 24 hours in advance. The increased predictability and the use of more of the available days allowed more precise and efficient scheduling, including a more even distribution of case time. However, this would probably also have had an impact with a “mixed” policy. A synergistic effect may have contributed, because the effects of each intervention could facilitate the successful implementation of the others. By reducing competition between elective and non-elective cases, dedicated ORs may increase availability and efficiency, which in itself reduces the incentives for non-clinical prioritization, thereby facilitating higher compliance with the new policies. Consensus-based interventions driven and run by the process-owners may also facilitate cooperation from staff and higher levels of compliance.

Despite the large growth in non-elective work (20%, p < 0.05), the proportions of non-elective surgery taking place at evening and night decreased from 30% to 27% and from 8% to 5%, respectively. Case time at night decreased by 26% (p < 0.05), and the number of nights without surgery increased from 55 to 112. Specifically, the proportion of out-of-hours case time was reduced for U2 and U3 cases. As cases requiring immediate attention always receive first priority and will be operated on as soon as possible at any hour [14], the reductions seen in the proportions of lower urgencies handled out-of-hours therefore reflects a decrease in the overflow cases. Efficiency improvements are more likely to manifest as improved OR accessibility for lower priority cases during the day [5,6]. Daytime surgery increased by over 48% (p < 0.05), and mean utilization of the dedicated ORs was 62%.

Median U2 waiting increased with 1.2 hours, but over 90% received treatment within MAWT in both periods. Median U3 waiting time was reduced with 12 hours, and the proportion of U3 cases treated within MAWT increased from 70% to 89%.

The analyses of U1 waiting time, however, indicate that issues such as skewing outliers should be considered. The number of U1 cases with excessive waiting was reduced in the second period. A number of factors have the potential to alter this measure, one of which is improved re-coding. While all non-elective patients are coded U1 upon hospital admittance by default, this is corrected once the level of urgency has been decided. The reduction in U1 cases with excessive waiting time, suggests that improved re-coding is largely responsible, by reducing the number of potential outliers. Improved re-coding may also have contributed to the distributional shift. The 1.5-hour reduction in median U1 waiting time is more likely to be caused by more time-critical emergencies in the second period than by improved efficiency.

The new booking system may have caused an artificial reduction in monitored waiting time. Booking an OR was not allowed until the patient was ready for surgery in the second period, which meant that pre-operative preparation time was excluded from monitored waiting time. Waiting time may therefore appear to be shorter. In addition to this, the new urgency classification as well as “gaming” and “delayed booking” could also affect monitored waiting time. It is therefore not possible to specify precisely the reduction in real waiting time.

### Overall results

The overall results indicate that the interventions allowed for a more effective use of daytime surgery and a more selective use of the ORs for high urgency patients during the night, while ensuring timely access to treatment. Not only is this cost-effective, but can also increase patient and staff satisfaction. Additional benefits are, for example, increased seniority of the surgeons and anaesthesiologists performing difficult cases [18,19], which also facilitates improved resident supervision and training opportunities, all of which may reduce errors and complications [20-22]. A more even distribution of case time throughout the day-, and evening shifts diminishes peaks and troughs, which is better for staff and patients, as it reduces stress and potential medical errors [22,23]. More emphasis should be put on these additional benefits when the cost efficiency of a redesign is evaluated.

### Limitations

There are several limitations to this study. First, as the data are from a single hospital the results may be difficult to generalize to other hospitals with different functional characteristics such as size, services provided, and case mix. However, it is reasonable to assume similar dynamics with regard to non-clinical prioritization may affect efficiency in other hospitals. Second, a before-and-after non randomized design was used, because randomization of patients would be impossible in this study. One possible, but not feasible alternative would have been to randomize several hospitals using dedicated or mixed ORs. Third, the study was retrospective, covering more than one intervention. There may also have been other factors influencing efficiency, which we did not assess. While we were not able to isolate and quantify the effects of the different policy changes, this could have been achieved by using other methods, such as discrete event simulation. Simulation models enable evaluation of different scenarios to measure the impact of different parameters. However, it may be difficult to include all relevant variables in a formal model. Therefore a before-and-after study based on observed effect is valuable even though it can be difficult – as in our case – to disentangle the effects of multiple changes made to the system.

### Future research

We propose future research focusing on: (1) analysis of health outcomes such as complications and mortality, and process outcomes that could adversely affect health outcomes, such as cancellation rates following a change in management policies; (2) investigations into whether such policy changes improve the estimation of future demand; (3) determination of the required level of demand that would justify the introduction of dedicated ORs and (4) development of generalizable algorithms for estimation of the number of dedicated ORs based on demand.

## Conclusions

Increasing demand for medical services requires appropriate allocation and effective use of OR resources while ensuring timely access to treatment. This study documents the impact of a new allocation strategy and policies for patient classification and OR booking on OR efficiency. The overall results indicate efficiency gains for both elective and the non-elective categories, with higher throughput and OR utilization, reduced overtime and waiting time, and no additional resources. The redesign facilitated effective daytime surgery and a more selective use of the ORs for high urgency patients during evenings and night. Reduced variability and higher predictability contributed to increased efficiency in the treatment of all patient groups. Although each of the interventions could improve efficiency, the synergistic effects of the redesign probably exceeded the sum of the individual effects of the new policies. Improvement, however, cannot be attained through new strategies alone; only modest change can be achieved without the support and cooperation of staff.

## Abbreviations

OR (s): Operating room (s); U1: U2 and U3, Urgency 1, 2 and 3; CT: Case time; MAWT: Maximum acceptable waiting time.

## Competing interests

The authors declare that they have no competing interests.

## Authors’ contributions

BS was the primary analyst and principal author of the paper. BH was involved in drafting, interpreting the analysis and reviewing the paper. OL participated in the data analysis and reviewed the paper. SF was involved in data collection, interpreting the analysis, and revising the paper. All authors read and approved the final paper.

## Pre-publication history

The pre-publication history for this paper can be accessed here:

http://www.biomedcentral.com/1472-6963/14/224/prepub
